# Sales trends of psychotropic drugs in the COVID-19 pandemic: A national database study in Brazil

**DOI:** 10.3389/fphar.2023.1131357

**Published:** 2023-03-17

**Authors:** Fernando de Sá Del Fiol, Cristiane de Cássia Bergamaschi, Luciane Cruz Lopes, Marcus Tolentino Silva, Silvio Barberato-Filho

**Affiliations:** Graduate Program in Pharmaceutical Sciences, University of Sorocaba, Sorocaba, Brazil

**Keywords:** psychotropics, antidepressants, COVID-19 pandemic, psycoactive substances, COVI-19 pandemic

## Abstract

**Background:** The social restrictions among coronavirus disease 2019 (COVID-19) pandemic have posed a thoughtful risk to mental health and have implications in the use of drugs, including antidepressants, anxiolytics and other psychotropics.

**Objective:** This study analyzed the sales data of the psychotropics prescribed in Brazil, in order to verify the change in consumption trends of these drugs during the COVID-19 pandemic.

**Methods:** This interrupted time-series analyzed psychotropic sales data, between January 2014 and July 2021, using the National System of Controlled Products Management from The Brazilian Health Regulatory Agency. The monthly mean DDDs per 1,000 inhabitants per day of psychotropic drugs was evaluated by analysis of variance (ANOVA) followed by Dunnett Multiple Comparisons Test. The changes in monthly trends in the use of the psychotropic studied were evaluated by Joinpoint regression.

**Results:** During the period studied, clonazepam, alprazolam, zolpidem and escitalopram were the most sold psychotropic drugs in Brazil. According to Joinpoint regression, an upward trend was observed in sales during the pandemic of pregabalin, escitalopram, lithium, desvenlafaxine, citalopram, buproprion and amitriptyline. An increase in psychotropic consumption was noted throughout the pandemic period, with the maximum consumption (2.61 DDDs) occurring in April 2021, with a downward trend in consumption that accompanied the drop in the number of deaths.

**Conclusions:** The increase in sales, mainly of antidepressants during the COVID-19 pandemic, draws attention to issues related to the mental health of the Brazilian population and on the need for greater monitoring in the dispensing of these drugs.

## Introduction

On 11 March 2020, the World Health Organization declared COVID-19, caused by the new Coronavirus, as a new global pandemic (Vindegaard and Benros, 2020). The new virus settles in the respiratory system, leading to cases of viral pneumonia, which is the main factor of morbidity related to the disease. Since the beginning of the pandemic, uncertainties regarding transmissibility, pathogenicity and lethality have taken hold of the scientific community and populations around the world, leading to feelings of apprehension, insecurity and fear (Castillo-Sánchez et al., 2022). World health agencies, in order to contain the pandemic, have established health protocols for the entire population, such as the use of alcoholic gel, masks, social isolation, *etc.* (Atalan, 2020) (Brodeur et al., 2021).

Modern civilization had never gone through a similar situation, when a part of it found itself “trapped” in their homes, avoiding contagion and infection or experiencing the early mourning of their family members. This naturally caused and continues to cause significant impacts on the mental health of everyone, whether infected or not (Brooks et al., 2020; Dubey et al., 2020; Ali et al., 2021; Castillo-Sánchez et al., 2022). On 25 February 2020, the first case of COVID-19 was confirmed in Brazil and, since then, there have been 24 million infected (January 2022 data), adding up to more than 600,000 deaths (Ministry, 2022).

The high mortality rates at the beginning of the pandemic, social isolation, associated with fear and insecurity due to lack of treatments; and/or the efficacy and availability of vaccines, led a large part of the population, including health professionals and young people (Wathelet et al., 2020; Meherali et al., 2021), to resort to chemical substances such as alcohol, illicit drugs and even psychotropic drugs in order to face this difficult situation (Garcia and Sanchez, 2020; Testino and Pellicano, 2020; Bennett et al., 2021; Levaillant et al., 2021; Madoz-Gúrpide et al., 2021; Søvold et al., 2021; Castillo-Sánchez et al., 2022; Estrela et al., 2022). Some studies have shown that the impact on the mental health of populations was important, with exacerbated conditions of depression, anxiety, panic, including suicidal ideation, especially among young people and health professionals (Gloster et al., 2020; de Vroege and van den Broek, 2021; Varga et al., 2021; Acharya et al., 2022; Appleby et al., 2022; Bozzola et al., 2022; Prudenzi et al., 2022).

In the United States, cases of depression and anxiety diagnosed among adults jumped from 36% to 41% between August 2020 and February 2021, reflecting the effects of the pandemic on this population (Vahratian et al., 2021). Likewise, visits to psychiatric offices also increased in the same period due to the pandemic (Holland et al., 2021). In Brazil, some studies have verified the prevalence and factors associated with depression or anxiety in health professionals or medical students and public-school teachers, during the COVID-19 pandemic. However, nationwide analyses of the sales profile of psychotropic drugs are still lacking in the literature.

In order to verify the impact of the COVID-19 pandemic on the mental health of the Brazilian population, this study used the national database on the dispensation of psychotropic drugs as an important resource for public health, since it can reflect the pattern of clinical and subclinical consumption of these drugs. Next, this study analyzed the sales data of psychotropic drugs prescribed in Brazil, in order to verify the consumption trend of these drugs during the COVID-19 pandemic.

## Materials and methods

### Study design

An interrupted time series was used to analyze the consumption trends of antidepressants and anxiolytics and other psychotropic medications (outcome of interest) during the COVID-19 pandemic (exposure of interest).

### Setting and study

Pharmacies and drugstores in Brazil have been required to register the number of all psychotropic medications sold monthly, since 2007, in the National System of Controlled Products Management database (known by the acronym SNGPC) from The Brazilian Health Regulatory Agency (ANVISA) (Varga et al., 2021). Monthly sales volume data were collected between January 2014 and July 2021.

### Data sources, measurement and variables

Data were publicly available on ANVISA website. The following variables were extracted: name of the active ingredient, trade name and respective presentations. The classification Anatomical Therapeutic Chemical (ATC) was used. Based on the commercial packaging of each psychotropic drug sold and the concentration of the active ingredient in each (e.g., pack of 10 pills of 20 mg each or pack of 30 pills of 10 mg each), the number of defined daily doses (DDDs)/1,000 inhabitants/day for each drug was calculated, as recommended by the World Health Organization (Acharya et al., 2022).

### Statistical analysis

Psychotropic drugs were described in average units of commercial presentations sold between 2014 and 2019 and during the pandemic (January 2020 and July 2021).

The monthly mean DDDs per 1,000 inhabitants per day of psychotropic drugs was evaluated by analysis of variance (ANOVA) followed by Dunnett Multiple Comparisons Test (Instat GraphPad Software version 3.05). The changes in monthly trends in the use of the psychotropic studied were evaluated by Joinpoint regression (Joinpoint Regression Program, version 4.9.0.0. Statistical Research and Applications Branch, National Cancer Institute). Pearson’s correlation coefficient verified the correlation between psychotropic consumption (expressed in DDDs/1,000 inhabitants/day) and the number of deaths in the same period. The level of statistical significance adopted was *p* < 0.05.

## Results

Table 1 described the 22 most sold psychotropic drugs in Brazil, between January 2014 and July 2021. The data shows the average amount of commercial presentations sold between January 2014 and December 2019 and during the pandemic (January 2020 and July 2021).

**TABLE 1:** Characterization and sales volume in Brazil of the studied psychotropics (January 2014 to July 2021).

Drugs	ATC code	DDD (mg)	Commercial presentations available in Brazil	Monthly commercial units sold (from 2014 January to December 2019)	Monthly commercial units sold in pandemic (January 2020 to July 2021)	Percentage of change during the pandemic (%)	*p*-value dunnett multiple comparisons test
Desvenlafaxine	N06AX23	50	95	128,257	376,340	193.43	<0.01
Pregabalin	N03AX16	300	101	247,499	566,553	128.91	<0.01
Quetiapine	N05AH04	400	147	387,505	768,629	98.35	<0.01
Zolpidem	N05CF02	10	67	622,704	1,180,424	89.56	<0.01
Escitalopram	N06AB10	10	167	600,022	1,105,859	84.30	<0.01
Lithium	N05AN01	889	20	114,617	198,031	72.78	<0.01
Sertraline	N06AB06	50	169	586,847	958,056	63.25	<0.01
Duloxetine	N06AX21	60	59	253,654	412,608	62.67	<0.01
Venlafaxine	N06AX16	100	129	313,622	498,415	58.92	<0.01
Trazodone	N06AX05	300	25	159,417	249,129	56.28	<0.01
Risperidone	N05AX08	5	143	313,277	471,593	50.54	<0.01
Amitriptyline	N06AA09	75	61	478,177	692,545	44.83	<0.01
Alprazolam	N05BA12	1	161	778,222	1,081,954	39.03	<0.01
Bupropion	N06AX12	300	66	172,191	236,244	37.20	<0.01
Paroxetine	N06AB05	20	105	327,938	446,277	36.09	<0.01
Fluoxetine	N06AB03	20	161	477,337	611,667	28.14	<0.01
Topiramate	N03AX11	300	102	167,985	214,237	27.53	<0.01
Clonazepam	N03AE01	8	98	1,591,101	1,772,727	11.42	NS
Citalopram	N06AB04	20	72	340,848	368,637	8.15	NS
Diazepam	N05BA01	10	75	254,338	240,858	−5.30	NS
Bromazepam	N05BA08	10	56	451,684	413,851	−8.38	NS
Lorazepam	N05BA06	2.5	42	219,716	185,807	−15.43	<0.01

ATC: anatomical therapeutic chemical; NS, non-significant (*p* > 0.05).

Between January 2014 and July 2021, clonazepam, zolpidem, alprazolam and escitalopram were the most sold psychotropics in Brazil. When comparing the pandemic years with previous years (2014–2019), there were statistically significant increases (*p* < 0.01) in almost all studied psychotropics (Table 1). The exceptions were Citalopram and benzodiazepines that did not show alterations. Lorazepam decreased by 15% during the studied period.

The data presented in Table 2 showed the consumption in DDD/1,000 inhabitants/day between 2014 and 2019 and during the pandemic, based on the historical series and on the level of significance related to the trend. There was an upward trend in sales of pregabalin, escitalopram, lithium, desvenlafaxine, citalopram, bupropion and amitriptyline.

**TABLE 2:** Consumption in DDDs between 2014 and 2019 and during the pandemic (January 2020 to July 2021).

Monthly average DDD/1,000 inhabitants/day	2014	2015	2016	2017	2018	2019	Pandemic (2020 January to July 2021)	Time series (*p*-value)	Trend (increase, decrease or no change)
Desvenlafaxine	0.39	0.47	0.55	0.68	1.23	1.9	2.72	<0.01	↑
Pregabalin	0.15	0.21	0.27	0.34	0.43	0.55	0.75	<0.01	↑
Lithium	0.21	0.24	0.28	0.31	0.32	0.42	0.51	<0.01	↑
Bupropion	0.48	0.55	0.58	0.64	0.73	0.85	0.96	<0.01	↑
Amitriptyline	0.51	0.57	0.66	0.95	0.89	0.79	1.06	<0.01	↑
Escitalopram	2.06	2.63	3.26	3.92	4.68	5.8	7,47	<0.05	↑
Citalopram	1.49	1.59	1.66	1.69	1.7	2.11	3.27	<0.05	↑
Clonazepam	1.23	1.32	1.42	1.55	1.56	1.59	1.67	<0.05	↓
Trazodone	0.16	0.18	0.21	0.25	0.29	0.34	0.39	<0.05	↓
Venlafaxine	0.86	1.01	1.18	1.37	1.54	1.82	2.09	<0.05	↓
Alprazolam	2.7	2.98	3.26	3.47	3.9	4.4	5.04	NS	↔
Zolpidem	0.95	1.24	1.58	2	2.58	3.34	4.35	NS	↔
Quetiapine	0.11	0.14	0.17	0.21	0.26	0.32	0.42	NS	↔
Risperidone	0.32	0.39	0.45	0.52	0.58	0.73	0.97	NS	↔
Duloxetine	0.44	0.63	0.81	1.02	1.24	1.48	1.75	NS	↔
Sertraline	1.98	2.3	2.64	2.97	3.3	4.08	4.93	NS	↔
Paroxetine	1.17	1.26	1.37	1.45	1.57	1.71	1.91	NS	↔
Fluoxetine	1.77	1.95	2.2	2.53	2.59	2.75	2.99	NS	↔
Topiramate	0.2	0.23	0.25	0.27	0.28	0.3	0.33	NS	↔
Diazepam	0.89	0.94	1.03	1.17	1.18	1.08	1.02	NS	↔
Bromazepam	0.96	0.95	0.94	0.9	0.88	0.86	0.83	NS	↔
Lorazepam	0.7	0.69	0.68	0.66	0.63	0.57	0.56	NS	↔

NS, non-significant (*p* > 0.05).

**FIGURE 1:**
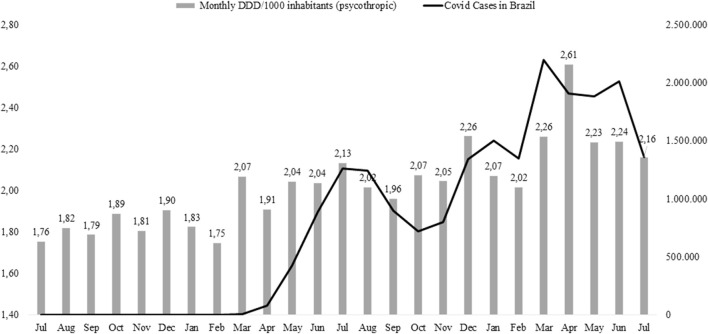
Monthly average (in DDDs) of psychotropic consumption in Brazil and number COVID-19 cases in Brazil (July 2019 to July 2021).

Figure 1 shows the average consumption in DDD/1,000 inhabitants/day of psychotropic drugs (bars) and the evolution in the number of COVID-19 cases in the country (line). It was observed that at the beginning of the pandemic (March 2020) and throughout its period, there was an increase in sales of psychotropic drugs. There was an increase from 1.75 DDDs/1,000 inhabitants/day in February 2020 to 2.07 DDDs/1,000 inhabitants/day in the following month. The same was observed when the number of deaths reaches its peak in the country (March 2021); in the following month (April 2021), the consumption of these drugs jumps from 2.26 DDDs/1,000 inhabitants/day to the maximum sale (2.61 DDDs/1,000 inhabitants/day). This drop in the consumption of psychotropic drugs accompanied the drop in the number of deaths caused by COVID-19.

**FIGURE 2:**
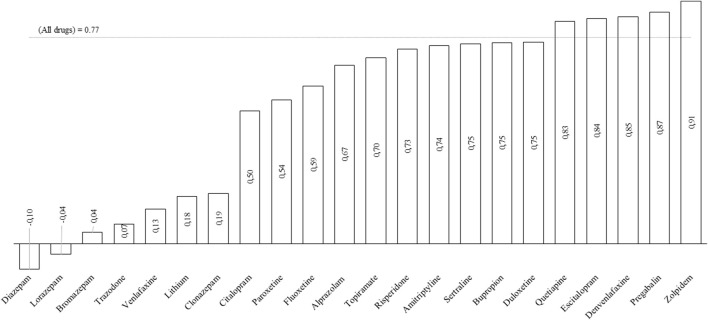
Pearson’s Correlation Coefficient of psychotropic and COVID-19 cases in Brazil during the pandemic (2020 January to 2021 July).

There was a strong correlation between the consumption of psychotropic drugs and cases of COVID-19 for most of psychotropics studied. For zolpidem there is very strong correlation (correlation coefficient: 0.91) (Figure 2).

## Discussion

During the pandemic period, there was an upward trend in sales of psychotropic drugs in Brazil, mainly; desvenlafaxine, pregabalin, quetiapine, zolpidem, escitalopram and lithium; compared to the period between January 2014 and December 2019, in which there were more sales of clonazepam, zolpidem, alprazolam and escitalopram. Between the period January 2014 to July 2021, the historical series showed an upward trend in sales of pregabalin, escitalopram, lithium, desvenlafaxine, citalopram, bupropion and amitriptyline.

The peak of the death toll in the country was in March 2021, when psychotropic consumption was at 2.26 DDDs/1,000 inhabitants/day. In the following month, consumption of these drugs jumped to 2.61 DDDs/1,000 inhabitants/day.

During the pandemic, a strong correlation was observed between the number of cases of deaths from COVID-19 in Brazil and the consumption of psychotropic drugs, especially zolpidem. Zolpidem is a hypnotic drug, non-benzodiazepine imidazopyridine with affinity to the α1 subunit of the GABA A receptor.

Another study conducted in the Brazilian capital (Escalante Saavedra et al., 2022) evaluated the impact of psychotropic consumption before and during the pandemic (2018–2020). The results showed Zolpidem consumption increasing from 6.2 Defined Daily Dose per 1,000 inhabitants-day (DHD) to 8.5 due to the COVID-19 Pandemic. Our data are very similar, showing an increase in Zolpidem consumption of around 89.5% (Table 1) in the studied period (2014–2021).

Milani et al. (2021), reported results similar to those found in the present study, showing an increase in the consumption of Z-hypnotics (zolpidem, zaleplon, and eszopiclone) among men and women in the United States and persisting in increased levels in the two waves of COVID-19 (Milani et al., 2021).

In the same sense, Levaillant et al. (2021), found even more alarming data in France, with a weekly increase of 2.5% in new users of hypnotics among young people (12–18 years old), during the pandemic period (Levaillant et al., 2021). In Portugal, a similar scenario was also observed during the pandemic, with a significant increase in the prescriptions of anxiolytics, hypnotics and sedatives, especially in the age group over 65 years (Estrela et al., 2022).

In France, researchers evaluated sleep habits in more than 1,000 patients and the pandemic’s interference in their habits. The authors reported that before the pandemic, only 12% of these patients were using hypnotic drugs. During the pandemic, due to the difficulties imposed on everyone, that number jumped to 41% (Beck et al., 2021). In Brazil, higher education students in the health area reported similar behaviors, with an increase of around 11% in the consumption of anxiolytics during the pandemic (Mata et al., 2021). Italy has also shown a sharp increase in the consumption of psychotropic drugs during the pandemic (Farina et al., 2021).

The reasons for the increase in psychotropic consumption seem very clear to us. In the United States, cases of depression increased about 3 times in the pandemic when compared to previous periods Certainly, the effects of the pandemic will not be restricted to the infectivity and pathogenicity of the virus, but will also leave important psychological consequences that, initially, have been treated with the use of psychotropic medications.

The reasons for the increase in psychotropic consumption seem very clear to us. In the United States, cases of depression increased about 3 times in the pandemic when compared to previous periods (Ettman et al., 2020). Recent studies in several countries around the world are unanimous in showing a significant increase in the consumption of psychotropic drugs during the period of the COVID-19 pandemic. Studies from Canada (Ying et al., 2023), France (Benistand et al., 2022; Sanchez et al., 2022), Portugal (Estrela et al., 2022), Scandinavia (Tiger et al., 2023), United States (Amill-Rosario et al., 2022), Danish (Bliddal et al., 2023) showed that the effects of the pandemic were not restricted to the infectivity and pathogenicity of the virus at the level of the respiratory system, but also left important psychological sequelae with increased used of psychotropic medications.

The increase in sales verified in Brazil in the present study, in all classes of psychotropic drugs, shows, in addition to the controlled clinical use of these drugs (through medical records), the sub-clinical use of drugs with high potential for abuse and reinforces the need for a closer look aware of the consumption of these drugs and the consequences of abuse and dependence associated with them.

## Conclusions

The increase in sales, mainly of antidepressants during the COVID-19 pandemic, draws attention to issues related to the mental health of the Brazilian population and makes us reflect on the need for greater monitoring in the dispensing of these drugs. Improving the national registration system for sales of psychotropic drugs could contribute with relevant information for the elaboration of public policies focused on mental health, seeking better guidance for personalized care and better doctor-patient decision making.

## Data Availability

Publicly available datasets were analyzed in this study. This data can be found here: https://dados.anvisa.gov.br/dados/SNGPC/Industrializados/
